# Supplementary material for: A Critical View of the Application of the APEX Software (Aqueous Photochemistry of Environmentally-Occurring Xenobiotics) to Predict Photoreaction Kinetics in Surface Freshwaters
Source: Molecules. 2019 Dec 18;25(1):9. doi: 10.3390/molecules25010009 (PMC7017383; doi:10.3390/molecules25010009)
Supplement: Supplementary file 1 [file molecules-25-00009-s001.zip › 3.2.4_gcc-4.4.0.zip!/3.2.4_gcc-4.4.0/3.2.4_gcc-4.4.0/doc/octave/html/liboctave/Copying.html]

Copying - Octave C++ Classes


Next: Introduction,
Previous: Acknowledgements,
Up: Top


---

## GNU GENERAL PUBLIC LICENSE

Version 3, 29 June 2007

```
     Copyright © 2007 Free Software Foundation, Inc. http://fsf.org/
     
     Everyone is permitted to copy and distribute verbatim copies of this
     license document, but changing it is not allowed.
```

### Preamble

The GNU General Public License is a free, copyleft license for
software and other kinds of works.

The licenses for most software and other practical works are designed
to take away your freedom to share and change the works. By contrast,
the GNU General Public License is intended to guarantee your freedom
to share and change all versions of a program—to make sure it remains
free software for all its users. We, the Free Software Foundation,
use the GNU General Public License for most of our software; it
applies also to any other work released this way by its authors. You
can apply it to your programs, too.

When we speak of free software, we are referring to freedom, not
price. Our General Public Licenses are designed to make sure that you
have the freedom to distribute copies of free software (and charge for
them if you wish), that you receive source code or can get it if you
want it, that you can change the software or use pieces of it in new
free programs, and that you know you can do these things.

To protect your rights, we need to prevent others from denying you
these rights or asking you to surrender the rights. Therefore, you
have certain responsibilities if you distribute copies of the
software, or if you modify it: responsibilities to respect the freedom
of others.

For example, if you distribute copies of such a program, whether
gratis or for a fee, you must pass on to the recipients the same
freedoms that you received. You must make sure that they, too,
receive or can get the source code. And you must show them these
terms so they know their rights.

Developers that use the GNU GPL protect your rights with two steps:
(1) assert copyright on the software, and (2) offer you this License
giving you legal permission to copy, distribute and/or modify it.

For the developers' and authors' protection, the GPL clearly explains
that there is no warranty for this free software. For both users' and
authors' sake, the GPL requires that modified versions be marked as
changed, so that their problems will not be attributed erroneously to
authors of previous versions.

Some devices are designed to deny users access to install or run
modified versions of the software inside them, although the
manufacturer can do so. This is fundamentally incompatible with the
aim of protecting users' freedom to change the software. The
systematic pattern of such abuse occurs in the area of products for
individuals to use, which is precisely where it is most unacceptable.
Therefore, we have designed this version of the GPL to prohibit the
practice for those products. If such problems arise substantially in
other domains, we stand ready to extend this provision to those
domains in future versions of the GPL, as needed to protect the
freedom of users.

Finally, every program is threatened constantly by software patents.
States should not allow patents to restrict development and use of
software on general-purpose computers, but in those that do, we wish
to avoid the special danger that patents applied to a free program
could make it effectively proprietary. To prevent this, the GPL
assures that patents cannot be used to render the program non-free.

The precise terms and conditions for copying, distribution and
modification follow.

### TERMS AND CONDITIONS

0. Definitions.

   “This License” refers to version 3 of the GNU General Public License.

   “Copyright” also means copyright-like laws that apply to other kinds
   of works, such as semiconductor masks.

   “The Program” refers to any copyrightable work licensed under this
   License. Each licensee is addressed as “you”. “Licensees” and
   “recipients” may be individuals or organizations.

   To “modify” a work means to copy from or adapt all or part of the work
   in a fashion requiring copyright permission, other than the making of
   an exact copy. The resulting work is called a “modified version” of
   the earlier work or a work “based on” the earlier work.

   A “covered work” means either the unmodified Program or a work based
   on the Program.

   To “propagate” a work means to do anything with it that, without
   permission, would make you directly or secondarily liable for
   infringement under applicable copyright law, except executing it on a
   computer or modifying a private copy. Propagation includes copying,
   distribution (with or without modification), making available to the
   public, and in some countries other activities as well.

   To “convey” a work means any kind of propagation that enables other
   parties to make or receive copies. Mere interaction with a user
   through a computer network, with no transfer of a copy, is not
   conveying.

   An interactive user interface displays “Appropriate Legal Notices” to
   the extent that it includes a convenient and prominently visible
   feature that (1) displays an appropriate copyright notice, and (2)
   tells the user that there is no warranty for the work (except to the
   extent that warranties are provided), that licensees may convey the
   work under this License, and how to view a copy of this License. If
   the interface presents a list of user commands or options, such as a
   menu, a prominent item in the list meets this criterion.- Source Code.

     The “source code” for a work means the preferred form of the work for
     making modifications to it. “Object code” means any non-source form
     of a work.

     A “Standard Interface” means an interface that either is an official
     standard defined by a recognized standards body, or, in the case of
     interfaces specified for a particular programming language, one that
     is widely used among developers working in that language.

     The “System Libraries” of an executable work include anything, other
     than the work as a whole, that (a) is included in the normal form of
     packaging a Major Component, but which is not part of that Major
     Component, and (b) serves only to enable use of the work with that
     Major Component, or to implement a Standard Interface for which an
     implementation is available to the public in source code form. A
     “Major Component”, in this context, means a major essential component
     (kernel, window system, and so on) of the specific operating system
     (if any) on which the executable work runs, or a compiler used to
     produce the work, or an object code interpreter used to run it.

     The “Corresponding Source” for a work in object code form means all
     the source code needed to generate, install, and (for an executable
     work) run the object code and to modify the work, including scripts to
     control those activities. However, it does not include the work's
     System Libraries, or general-purpose tools or generally available free
     programs which are used unmodified in performing those activities but
     which are not part of the work. For example, Corresponding Source
     includes interface definition files associated with source files for
     the work, and the source code for shared libraries and dynamically
     linked subprograms that the work is specifically designed to require,
     such as by intimate data communication or control flow between those
     subprograms and other parts of the work.

     The Corresponding Source need not include anything that users can
     regenerate automatically from other parts of the Corresponding Source.

     The Corresponding Source for a work in source code form is that same
     work.- Basic Permissions.

       All rights granted under this License are granted for the term of
       copyright on the Program, and are irrevocable provided the stated
       conditions are met. This License explicitly affirms your unlimited
       permission to run the unmodified Program. The output from running a
       covered work is covered by this License only if the output, given its
       content, constitutes a covered work. This License acknowledges your
       rights of fair use or other equivalent, as provided by copyright law.

       You may make, run and propagate covered works that you do not convey,
       without conditions so long as your license otherwise remains in force.
       You may convey covered works to others for the sole purpose of having
       them make modifications exclusively for you, or provide you with
       facilities for running those works, provided that you comply with the
       terms of this License in conveying all material for which you do not
       control copyright. Those thus making or running the covered works for
       you must do so exclusively on your behalf, under your direction and
       control, on terms that prohibit them from making any copies of your
       copyrighted material outside their relationship with you.

       Conveying under any other circumstances is permitted solely under the
       conditions stated below. Sublicensing is not allowed; section 10
       makes it unnecessary.- Protecting Users' Legal Rights From Anti-Circumvention Law.

         No covered work shall be deemed part of an effective technological
         measure under any applicable law fulfilling obligations under article
         11 of the WIPO copyright treaty adopted on 20 December 1996, or
         similar laws prohibiting or restricting circumvention of such
         measures.

         When you convey a covered work, you waive any legal power to forbid
         circumvention of technological measures to the extent such
         circumvention is effected by exercising rights under this License with
         respect to the covered work, and you disclaim any intention to limit
         operation or modification of the work as a means of enforcing, against
         the work's users, your or third parties' legal rights to forbid
         circumvention of technological measures.- Conveying Verbatim Copies.

           You may convey verbatim copies of the Program's source code as you
           receive it, in any medium, provided that you conspicuously and
           appropriately publish on each copy an appropriate copyright notice;
           keep intact all notices stating that this License and any
           non-permissive terms added in accord with section 7 apply to the code;
           keep intact all notices of the absence of any warranty; and give all
           recipients a copy of this License along with the Program.

           You may charge any price or no price for each copy that you convey,
           and you may offer support or warranty protection for a fee.- Conveying Modified Source Versions.

             You may convey a work based on the Program, or the modifications to
             produce it from the Program, in the form of source code under the
             terms of section 4, provided that you also meet all of these
             conditions:
             1. The work must carry prominent notices stating that you modified it,
                and giving a relevant date.- The work must carry prominent notices stating that it is released
                  under this License and any conditions added under section 7. This
                  requirement modifies the requirement in section 4 to “keep intact all
                  notices”.- You must license the entire work, as a whole, under this License to
                    anyone who comes into possession of a copy. This License will
                    therefore apply, along with any applicable section 7 additional terms,
                    to the whole of the work, and all its parts, regardless of how they
                    are packaged. This License gives no permission to license the work in
                    any other way, but it does not invalidate such permission if you have
                    separately received it.- If the work has interactive user interfaces, each must display
                      Appropriate Legal Notices; however, if the Program has interactive
                      interfaces that do not display Appropriate Legal Notices, your work
                      need not make them do so.

             A compilation of a covered work with other separate and independent
             works, which are not by their nature extensions of the covered work,
             and which are not combined with it such as to form a larger program,
             in or on a volume of a storage or distribution medium, is called an
             “aggregate” if the compilation and its resulting copyright are not
             used to limit the access or legal rights of the compilation's users
             beyond what the individual works permit. Inclusion of a covered work
             in an aggregate does not cause this License to apply to the other
             parts of the aggregate.- Conveying Non-Source Forms.

               You may convey a covered work in object code form under the terms of
               sections 4 and 5, provided that you also convey the machine-readable
               Corresponding Source under the terms of this License, in one of these
               ways:
               1. Convey the object code in, or embodied in, a physical product
                  (including a physical distribution medium), accompanied by the
                  Corresponding Source fixed on a durable physical medium customarily
                  used for software interchange.- Convey the object code in, or embodied in, a physical product
                    (including a physical distribution medium), accompanied by a written
                    offer, valid for at least three years and valid for as long as you
                    offer spare parts or customer support for that product model, to give
                    anyone who possesses the object code either (1) a copy of the
                    Corresponding Source for all the software in the product that is
                    covered by this License, on a durable physical medium customarily used
                    for software interchange, for a price no more than your reasonable
                    cost of physically performing this conveying of source, or (2) access
                    to copy the Corresponding Source from a network server at no charge.- Convey individual copies of the object code with a copy of the written
                      offer to provide the Corresponding Source. This alternative is
                      allowed only occasionally and noncommercially, and only if you
                      received the object code with such an offer, in accord with subsection
                      6b.- Convey the object code by offering access from a designated place
                        (gratis or for a charge), and offer equivalent access to the
                        Corresponding Source in the same way through the same place at no
                        further charge. You need not require recipients to copy the
                        Corresponding Source along with the object code. If the place to copy
                        the object code is a network server, the Corresponding Source may be
                        on a different server (operated by you or a third party) that supports
                        equivalent copying facilities, provided you maintain clear directions
                        next to the object code saying where to find the Corresponding Source.
                        Regardless of what server hosts the Corresponding Source, you remain
                        obligated to ensure that it is available for as long as needed to
                        satisfy these requirements.- Convey the object code using peer-to-peer transmission, provided you
                          inform other peers where the object code and Corresponding Source of
                          the work are being offered to the general public at no charge under
                          subsection 6d.

               A separable portion of the object code, whose source code is excluded
               from the Corresponding Source as a System Library, need not be
               included in conveying the object code work.

               A “User Product” is either (1) a “consumer product”, which means any
               tangible personal property which is normally used for personal,
               family, or household purposes, or (2) anything designed or sold for
               incorporation into a dwelling. In determining whether a product is a
               consumer product, doubtful cases shall be resolved in favor of
               coverage. For a particular product received by a particular user,
               “normally used” refers to a typical or common use of that class of
               product, regardless of the status of the particular user or of the way
               in which the particular user actually uses, or expects or is expected
               to use, the product. A product is a consumer product regardless of
               whether the product has substantial commercial, industrial or
               non-consumer uses, unless such uses represent the only significant
               mode of use of the product.

               “Installation Information” for a User Product means any methods,
               procedures, authorization keys, or other information required to
               install and execute modified versions of a covered work in that User
               Product from a modified version of its Corresponding Source. The
               information must suffice to ensure that the continued functioning of
               the modified object code is in no case prevented or interfered with
               solely because modification has been made.

               If you convey an object code work under this section in, or with, or
               specifically for use in, a User Product, and the conveying occurs as
               part of a transaction in which the right of possession and use of the
               User Product is transferred to the recipient in perpetuity or for a
               fixed term (regardless of how the transaction is characterized), the
               Corresponding Source conveyed under this section must be accompanied
               by the Installation Information. But this requirement does not apply
               if neither you nor any third party retains the ability to install
               modified object code on the User Product (for example, the work has
               been installed in ROM).

               The requirement to provide Installation Information does not include a
               requirement to continue to provide support service, warranty, or
               updates for a work that has been modified or installed by the
               recipient, or for the User Product in which it has been modified or
               installed. Access to a network may be denied when the modification
               itself materially and adversely affects the operation of the network
               or violates the rules and protocols for communication across the
               network.

               Corresponding Source conveyed, and Installation Information provided,
               in accord with this section must be in a format that is publicly
               documented (and with an implementation available to the public in
               source code form), and must require no special password or key for
               unpacking, reading or copying.- Additional Terms.

                 “Additional permissions” are terms that supplement the terms of this
                 License by making exceptions from one or more of its conditions.
                 Additional permissions that are applicable to the entire Program shall
                 be treated as though they were included in this License, to the extent
                 that they are valid under applicable law. If additional permissions
                 apply only to part of the Program, that part may be used separately
                 under those permissions, but the entire Program remains governed by
                 this License without regard to the additional permissions.

                 When you convey a copy of a covered work, you may at your option
                 remove any additional permissions from that copy, or from any part of
                 it. (Additional permissions may be written to require their own
                 removal in certain cases when you modify the work.) You may place
                 additional permissions on material, added by you to a covered work,
                 for which you have or can give appropriate copyright permission.

                 Notwithstanding any other provision of this License, for material you
                 add to a covered work, you may (if authorized by the copyright holders
                 of that material) supplement the terms of this License with terms:
                 1. Disclaiming warranty or limiting liability differently from the terms
                    of sections 15 and 16 of this License; or- Requiring preservation of specified reasonable legal notices or author
                      attributions in that material or in the Appropriate Legal Notices
                      displayed by works containing it; or- Prohibiting misrepresentation of the origin of that material, or
                        requiring that modified versions of such material be marked in
                        reasonable ways as different from the original version; or- Limiting the use for publicity purposes of names of licensors or
                          authors of the material; or- Declining to grant rights under trademark law for use of some trade
                            names, trademarks, or service marks; or- Requiring indemnification of licensors and authors of that material by
                              anyone who conveys the material (or modified versions of it) with
                              contractual assumptions of liability to the recipient, for any
                              liability that these contractual assumptions directly impose on those
                              licensors and authors.

                 All other non-permissive additional terms are considered “further
                 restrictions” within the meaning of section 10. If the Program as you
                 received it, or any part of it, contains a notice stating that it is
                 governed by this License along with a term that is a further
                 restriction, you may remove that term. If a license document contains
                 a further restriction but permits relicensing or conveying under this
                 License, you may add to a covered work material governed by the terms
                 of that license document, provided that the further restriction does
                 not survive such relicensing or conveying.

                 If you add terms to a covered work in accord with this section, you
                 must place, in the relevant source files, a statement of the
                 additional terms that apply to those files, or a notice indicating
                 where to find the applicable terms.

                 Additional terms, permissive or non-permissive, may be stated in the
                 form of a separately written license, or stated as exceptions; the
                 above requirements apply either way.- Termination.

                   You may not propagate or modify a covered work except as expressly
                   provided under this License. Any attempt otherwise to propagate or
                   modify it is void, and will automatically terminate your rights under
                   this License (including any patent licenses granted under the third
                   paragraph of section 11).

                   However, if you cease all violation of this License, then your license
                   from a particular copyright holder is reinstated (a) provisionally,
                   unless and until the copyright holder explicitly and finally
                   terminates your license, and (b) permanently, if the copyright holder
                   fails to notify you of the violation by some reasonable means prior to
                   60 days after the cessation.

                   Moreover, your license from a particular copyright holder is
                   reinstated permanently if the copyright holder notifies you of the
                   violation by some reasonable means, this is the first time you have
                   received notice of violation of this License (for any work) from that
                   copyright holder, and you cure the violation prior to 30 days after
                   your receipt of the notice.

                   Termination of your rights under this section does not terminate the
                   licenses of parties who have received copies or rights from you under
                   this License. If your rights have been terminated and not permanently
                   reinstated, you do not qualify to receive new licenses for the same
                   material under section 10.- Acceptance Not Required for Having Copies.

                     You are not required to accept this License in order to receive or run
                     a copy of the Program. Ancillary propagation of a covered work
                     occurring solely as a consequence of using peer-to-peer transmission
                     to receive a copy likewise does not require acceptance. However,
                     nothing other than this License grants you permission to propagate or
                     modify any covered work. These actions infringe copyright if you do
                     not accept this License. Therefore, by modifying or propagating a
                     covered work, you indicate your acceptance of this License to do so.- Automatic Licensing of Downstream Recipients.

                       Each time you convey a covered work, the recipient automatically
                       receives a license from the original licensors, to run, modify and
                       propagate that work, subject to this License. You are not responsible
                       for enforcing compliance by third parties with this License.

                       An “entity transaction” is a transaction transferring control of an
                       organization, or substantially all assets of one, or subdividing an
                       organization, or merging organizations. If propagation of a covered
                       work results from an entity transaction, each party to that
                       transaction who receives a copy of the work also receives whatever
                       licenses to the work the party's predecessor in interest had or could
                       give under the previous paragraph, plus a right to possession of the
                       Corresponding Source of the work from the predecessor in interest, if
                       the predecessor has it or can get it with reasonable efforts.

                       You may not impose any further restrictions on the exercise of the
                       rights granted or affirmed under this License. For example, you may
                       not impose a license fee, royalty, or other charge for exercise of
                       rights granted under this License, and you may not initiate litigation
                       (including a cross-claim or counterclaim in a lawsuit) alleging that
                       any patent claim is infringed by making, using, selling, offering for
                       sale, or importing the Program or any portion of it.- Patents.

                         A “contributor” is a copyright holder who authorizes use under this
                         License of the Program or a work on which the Program is based. The
                         work thus licensed is called the contributor's “contributor version”.

                         A contributor's “essential patent claims” are all patent claims owned
                         or controlled by the contributor, whether already acquired or
                         hereafter acquired, that would be infringed by some manner, permitted
                         by this License, of making, using, or selling its contributor version,
                         but do not include claims that would be infringed only as a
                         consequence of further modification of the contributor version. For
                         purposes of this definition, “control” includes the right to grant
                         patent sublicenses in a manner consistent with the requirements of
                         this License.

                         Each contributor grants you a non-exclusive, worldwide, royalty-free
                         patent license under the contributor's essential patent claims, to
                         make, use, sell, offer for sale, import and otherwise run, modify and
                         propagate the contents of its contributor version.

                         In the following three paragraphs, a “patent license” is any express
                         agreement or commitment, however denominated, not to enforce a patent
                         (such as an express permission to practice a patent or covenant not to
                         sue for patent infringement). To “grant” such a patent license to a
                         party means to make such an agreement or commitment not to enforce a
                         patent against the party.

                         If you convey a covered work, knowingly relying on a patent license,
                         and the Corresponding Source of the work is not available for anyone
                         to copy, free of charge and under the terms of this License, through a
                         publicly available network server or other readily accessible means,
                         then you must either (1) cause the Corresponding Source to be so
                         available, or (2) arrange to deprive yourself of the benefit of the
                         patent license for this particular work, or (3) arrange, in a manner
                         consistent with the requirements of this License, to extend the patent
                         license to downstream recipients. “Knowingly relying” means you have
                         actual knowledge that, but for the patent license, your conveying the
                         covered work in a country, or your recipient's use of the covered work
                         in a country, would infringe one or more identifiable patents in that
                         country that you have reason to believe are valid.

                         If, pursuant to or in connection with a single transaction or
                         arrangement, you convey, or propagate by procuring conveyance of, a
                         covered work, and grant a patent license to some of the parties
                         receiving the covered work authorizing them to use, propagate, modify
                         or convey a specific copy of the covered work, then the patent license
                         you grant is automatically extended to all recipients of the covered
                         work and works based on it.

                         A patent license is “discriminatory” if it does not include within the
                         scope of its coverage, prohibits the exercise of, or is conditioned on
                         the non-exercise of one or more of the rights that are specifically
                         granted under this License. You may not convey a covered work if you
                         are a party to an arrangement with a third party that is in the
                         business of distributing software, under which you make payment to the
                         third party based on the extent of your activity of conveying the
                         work, and under which the third party grants, to any of the parties
                         who would receive the covered work from you, a discriminatory patent
                         license (a) in connection with copies of the covered work conveyed by
                         you (or copies made from those copies), or (b) primarily for and in
                         connection with specific products or compilations that contain the
                         covered work, unless you entered into that arrangement, or that patent
                         license was granted, prior to 28 March 2007.

                         Nothing in this License shall be construed as excluding or limiting
                         any implied license or other defenses to infringement that may
                         otherwise be available to you under applicable patent law.- No Surrender of Others' Freedom.

                           If conditions are imposed on you (whether by court order, agreement or
                           otherwise) that contradict the conditions of this License, they do not
                           excuse you from the conditions of this License. If you cannot convey
                           a covered work so as to satisfy simultaneously your obligations under
                           this License and any other pertinent obligations, then as a
                           consequence you may not convey it at all. For example, if you agree
                           to terms that obligate you to collect a royalty for further conveying
                           from those to whom you convey the Program, the only way you could
                           satisfy both those terms and this License would be to refrain entirely
                           from conveying the Program.- Use with the GNU Affero General Public License.

                             Notwithstanding any other provision of this License, you have
                             permission to link or combine any covered work with a work licensed
                             under version 3 of the GNU Affero General Public License into a single
                             combined work, and to convey the resulting work. The terms of this
                             License will continue to apply to the part which is the covered work,
                             but the special requirements of the GNU Affero General Public License,
                             section 13, concerning interaction through a network will apply to the
                             combination as such.- Revised Versions of this License.

                               The Free Software Foundation may publish revised and/or new versions
                               of the GNU General Public License from time to time. Such new
                               versions will be similar in spirit to the present version, but may
                               differ in detail to address new problems or concerns.

                               Each version is given a distinguishing version number. If the Program
                               specifies that a certain numbered version of the GNU General Public
                               License “or any later version” applies to it, you have the option of
                               following the terms and conditions either of that numbered version or
                               of any later version published by the Free Software Foundation. If
                               the Program does not specify a version number of the GNU General
                               Public License, you may choose any version ever published by the Free
                               Software Foundation.

                               If the Program specifies that a proxy can decide which future versions
                               of the GNU General Public License can be used, that proxy's public
                               statement of acceptance of a version permanently authorizes you to
                               choose that version for the Program.

                               Later license versions may give you additional or different
                               permissions. However, no additional obligations are imposed on any
                               author or copyright holder as a result of your choosing to follow a
                               later version.- Disclaimer of Warranty.

                                 THERE IS NO WARRANTY FOR THE PROGRAM, TO THE EXTENT PERMITTED BY
                                 APPLICABLE LAW. EXCEPT WHEN OTHERWISE STATED IN WRITING THE COPYRIGHT
                                 HOLDERS AND/OR OTHER PARTIES PROVIDE THE PROGRAM “AS IS” WITHOUT
                                 WARRANTY OF ANY KIND, EITHER EXPRESSED OR IMPLIED, INCLUDING, BUT NOT
                                 LIMITED TO, THE IMPLIED WARRANTIES OF MERCHANTABILITY AND FITNESS FOR
                                 A PARTICULAR PURPOSE. THE ENTIRE RISK AS TO THE QUALITY AND
                                 PERFORMANCE OF THE PROGRAM IS WITH YOU. SHOULD THE PROGRAM PROVE
                                 DEFECTIVE, YOU ASSUME THE COST OF ALL NECESSARY SERVICING, REPAIR OR
                                 CORRECTION.- Limitation of Liability.

                                   IN NO EVENT UNLESS REQUIRED BY APPLICABLE LAW OR AGREED TO IN WRITING
                                   WILL ANY COPYRIGHT HOLDER, OR ANY OTHER PARTY WHO MODIFIES AND/OR
                                   CONVEYS THE PROGRAM AS PERMITTED ABOVE, BE LIABLE TO YOU FOR DAMAGES,
                                   INCLUDING ANY GENERAL, SPECIAL, INCIDENTAL OR CONSEQUENTIAL DAMAGES
                                   ARISING OUT OF THE USE OR INABILITY TO USE THE PROGRAM (INCLUDING BUT
                                   NOT LIMITED TO LOSS OF DATA OR DATA BEING RENDERED INACCURATE OR
                                   LOSSES SUSTAINED BY YOU OR THIRD PARTIES OR A FAILURE OF THE PROGRAM
                                   TO OPERATE WITH ANY OTHER PROGRAMS), EVEN IF SUCH HOLDER OR OTHER
                                   PARTY HAS BEEN ADVISED OF THE POSSIBILITY OF SUCH DAMAGES.- Interpretation of Sections 15 and 16.

                                     If the disclaimer of warranty and limitation of liability provided
                                     above cannot be given local legal effect according to their terms,
                                     reviewing courts shall apply local law that most closely approximates
                                     an absolute waiver of all civil liability in connection with the
                                     Program, unless a warranty or assumption of liability accompanies a
                                     copy of the Program in return for a fee.

### END OF TERMS AND CONDITIONS

### How to Apply These Terms to Your New Programs

If you develop a new program, and you want it to be of the greatest
possible use to the public, the best way to achieve this is to make it
free software which everyone can redistribute and change under these
terms.

To do so, attach the following notices to the program. It is safest
to attach them to the start of each source file to most effectively
state the exclusion of warranty; and each file should have at least
the “copyright” line and a pointer to where the full notice is found.

```
     one line to give the program's name and a brief idea of what it does.
     Copyright (C) year name of author
     
     This program is free software: you can redistribute it and/or modify
     it under the terms of the GNU General Public License as published by
     the Free Software Foundation, either version 3 of the License, or (at
     your option) any later version.
     
     This program is distributed in the hope that it will be useful, but
     WITHOUT ANY WARRANTY; without even the implied warranty of
     MERCHANTABILITY or FITNESS FOR A PARTICULAR PURPOSE.  See the GNU
     General Public License for more details.
     
     You should have received a copy of the GNU General Public License
     along with this program.  If not, see http://www.gnu.org/licenses/.
```

Also add information on how to contact you by electronic and paper mail.

If the program does terminal interaction, make it output a short
notice like this when it starts in an interactive mode:

```
     program Copyright (C) year name of author
     This program comes with ABSOLUTELY NO WARRANTY; for details type show w.
     This is free software, and you are welcome to redistribute it
     under certain conditions; type show c for details.
```

The hypothetical commands show w and show c should show
the appropriate parts of the General Public License. Of course, your
program's commands might be different; for a GUI interface, you would
use an “about box”.

You should also get your employer (if you work as a programmer) or school,
if any, to sign a “copyright disclaimer” for the program, if necessary.
For more information on this, and how to apply and follow the GNU GPL, see
http://www.gnu.org/licenses/.

The GNU General Public License does not permit incorporating your
program into proprietary programs. If your program is a subroutine
library, you may consider it more useful to permit linking proprietary
applications with the library. If this is what you want to do, use
the GNU Lesser General Public License instead of this License. But
first, please read http://www.gnu.org/philosophy/why-not-lgpl.html.
